# Contact Tracing of Tuberculosis: A Systematic Review of Transmission Modelling Studies

**DOI:** 10.1371/journal.pone.0072470

**Published:** 2013-09-04

**Authors:** Matt Begun, Anthony T. Newall, Guy B. Marks, James G. Wood

**Affiliations:** 1 School of Public Health and Community Medicine, Faculty of Medicine, University of New South Wales, Sydney, Australia; 2 Respiratory and Environmental Epidemiology, Woolcock Institute of Medical Research and Liverpool Hospital, Sydney, Australia; Massey University, New Zealand

## Abstract

The WHO recommended intervention of Directly Observed Treatment, Short-course (DOTS) appears to have been less successful than expected in reducing the burden of TB in some high prevalence settings. One strategy for enhancing DOTS is incorporating active case-finding through screening contacts of TB patients as widely used in low-prevalence settings. Predictive models that incorporate population-level effects on transmission provide one means of predicting impacts of such interventions. We aim to identify all TB transmission modelling studies addressing contact tracing and to describe and critically assess their modelling assumptions, parameter choices and relevance to policy. We searched MEDLINE, SCOPUS, COMPENDEX, Google Scholar and Web of Science databases for relevant English language publications up to February 2012. Of the 1285 studies identified, only 5 studies met our inclusion criteria of models of TB transmission dynamics in human populations designed to incorporate contact tracing as an intervention. Detailed implementation of contact processes was only present in two studies, while only one study presented a model for a high prevalence, developing world setting. Some use of relevant data for parameter estimation was made in each study however validation of the predicted impact of interventions was not attempted in any of the studies. Despite a large body of literature on TB transmission modelling, few published studies incorporate contact tracing. There is considerable scope for future analyses to make better use of data and to apply individual based models to facilitate more realistic patterns of infectious contact. Combined with a focus on high burden settings this would greatly increase the potential for models to inform the use of contract tracing as a TB control policy. Our findings highlight the potential for collaborative work between clinicians, epidemiologists and modellers to gather data required to enhance model development and validation and hence better inform future public health policy.

## Introduction

Tuberculosis (TB) is among the world's leading infectious causes of death, ranked second only to HIV/AIDS in mortality due to a single infectious agent [1]. The WHO estimates that in 2011 there were 1.4 million deaths from TB and 8.7 million new cases [2]. While TB has largely been controlled in the developed world, control efforts have been less successful in Africa, Asia and parts of Eastern Europe. The WHO estimates that over 95% of cases and deaths occur in developing countries [1].

The WHO reports that the Millennium and Stop TB Partnership [3] targets for incidence and mortality reduction could be met by 2015 for the global population [4] based on current global trends. However the incidence target is unlikely to be reached in the South East Asian region and the mortality targets are unlikely to be reached in the African region [5], [6]. Directly Observed Treatment, Short-course (DOTS), the internationally recommended program established to reach these targets [7], does not appear to have been as successful as expected in some high prevalence settings. A recent study in Vietnam found that the prevalence of TB was 1.6 times higher than previously estimated by WHO [8].

Active case finding provides a promising addition to the passive case finding approach of DOTS. Active case finding approaches include screening high risk groups and contact tracing to increase the rate of TB case identification. Finding and screening case-contacts may be a very effective method of increasing case detection rates [9]. The goal of contact tracing is to reduce the time required to detect and treat a case and hence reduce the ability of infectious patients to transmit the disease. While contact tracing has been used extensively as a control strategy for TB in the developed world (typically low prevalence settings) it is uncommon in developing countries with high prevalence. Very few randomized controlled trials (RCTs) have specifically examined the effect of active case finding among contacts of patients with microbiologically proven pulmonary TB on case detection rates [10].

Disease transmission models are frequently used to understand epidemic dynamics at a population level for a variety of communicable diseases [11]. They can also be used to help inform researchers about additional data needed to better inform policy and future studies. Perhaps most importantly, they can be used to make predictions about the likely impact of competing policy options for disease prevention in a very cost-effective manner, reducing the need to run expensive RCTs in different settings for each option. This often involves the integration of data from a variety of sources to make predictions about future incidence and the effectiveness of interventions on reducing incidence.

Models were first used to study the dynamics of TB epidemics in the 1960s [12] and have been used extensively since the mid 1990s [13]. Models of TB have been used to describe the epidemiology of the disease [14], as a tool for evaluating the impact and cost-effectiveness of interventions [15], [16], and for describing the role that population structure plays on the dynamics of an epidemic [17], [18]. For broader review of mathematical models of TB see the papers by Colijn et al. [16] or Castillo-Chavez and Song [13].

Most models of TB dynamics in the literature are variants of a compartmental model structure where the host population is divided into mutually-exclusive classes (or compartments) based on their stage of infection – Susceptible (not infected), Exposed or Latent (infected but without active disease), Infectious (active disease) and Recovered (SEIR). The transitions an individual may take between these compartments and the rates at which they do so are typically represented as a series of ordinary differential equations that depend on parameters that summarise observed TB epidemiology.

The aim of this review is to identify all TB transmission modelling studies addressing contact tracing as an intervention and to describe and critically assess their modelling assumptions, parameter choices and relevance to policy. By doing so, we hope to better inform future modelling efforts and summarise current findings from models on the value of contact tracing as a public health intervention.

## Methods

### Search strategy

We searched MEDLINE, SCOPUS, COMPENDEX, Google Scholar and Web of Science databases for studies presenting TB transmission models of human populations with contact tracing as an intervention. We limited our search to relevant English language publications from earliest date to February 2012 inclusive. For MEDLINE the search terms used were “Humans” AND (“tuberculosis” OR “latent tuberculosis” OR “tuberculosis, multidrug-resistant” OR “tuberculosis, pulmonary”) AND (“models, theoretical” OR “models, biological” OR “nonlinear dynamics”). For the other databases, which did not use hierarchical keyword structures, title searches on (“TB” OR “Tuberculosis”) and “model” were used. Titles and abstracts were screened to identify studies presenting models of TB transmission dynamics in human populations. Abstracts were then reviewed to limit results to models incorporating active case finding, population stratification or that used a heterogeneous contact structure. Full-text articles were obtained and articles were included for review if they presented models designed to incorporate contact tracing as an intervention. While a number of articles present similar models to those included, models that did not explicitly discuss contact tracing were excluded.

### Search results

**Figure 1:**
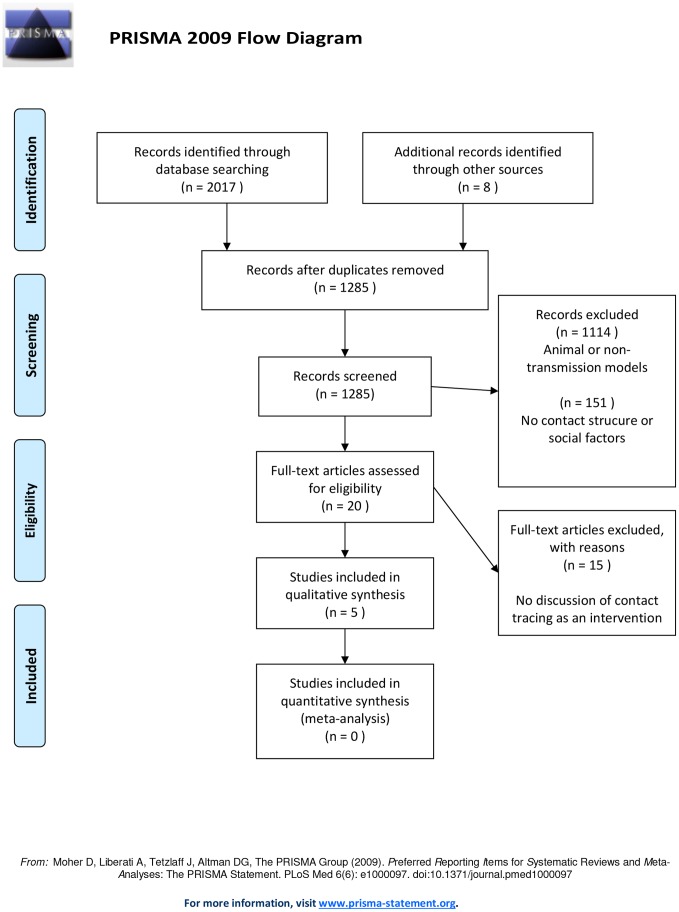
Flow chart of document search.

Of the 1285 studies identified through searching, 1114 were excluded by title search, 151 were excluded through abstract search and 15 were excluded through full text search. Five studies met our inclusion criteria and were included in the review (see Figure 1).

## Results

A basic summary of each of the studies reviewed can be found in Table 1.

**Table 1:** Summary of key characteristics of studies under review.

Author	Guzzetta et al.	Mellor et al.	Tian et al.	Aparicio et al.	Ziv et al.
Year	2011	2011	2011	2006	2001
**Model Type**	Individual Based Model (Stochastic)	Discrete Event (Stochastic)	Systems Dynamics (Deterministic)	Compartment (Deterministic)	Compartment (Deterministic)
**Contact Structure**	Multiple clusters – household, work, school, etc.	Clustered households	Homogeneous	Homogeneous	Homogeneous
**Model Implementation Of** **Contact Structure**	Spatial network structure	Clustering of HIV and TB infections	N/A	N/A	N/A
**Other Modelling Elements**	Reactivation, re- infection, spatial effects, age	HIV, age, gender, Fast/Slow Latency, Re-infection, Non- infectious tracking	Parallel classes for investigated and un- investigated cases	Primary/Latent exposure classes	Early/Late Latent classes
**Interventions**	Contact tracing proposed but not implemented	Contact Tracing, Targeted active case finding (HIV)	Contact Tracing (approx.)	Contact Tracing (approx.) Screening	Contact Tracing (approx.) Screening
**Implementation Of** **Contact Tracing**	N/A	Direct simulation	Transition rates between un-investigated and investigated compartments	Increased treatment rates for latent TB	Increased treatment rates for latent and active TB
**Setting**	Low prevalence	High prevalence	Low prevalence	Low prevalance	Theoretical
**Region**	USA	Africa	Canada	USA	Theoretical
**Lifespan**	80 years	Calculated from life tables	37 years	Varies (50–110 approx.)	50 years
**Constant Population**	Yes	Yes	No	No	No
**Transmissibility**	Varies	10 per person per year (1 in household)	18.8 per person per year	Varies	7 per person per year
**Mean Survival Time with TB**	7.5 years	3.3 years without HIV 0.3 years with HIV	27 years	10 years	7.2 years
**Duration of infectious** **period**	0.3 years*	2.0 years	0.5 years	0.5 years	1.5 years†
**Sensitivity Analysis**	Variables which could not be directly estimated from data	Analysis conducted for HIV prevalence only	Coefficient and mean time of tracing contacts	Variables which could not be directly estimated from data	None†
**High Influence Parameters**	N/A	HIV prevalence	Contact detection rate	N/A	N/A
**Validation**	Population compared to US census and CDC data	Household and community transmission rates, TB incidence, HIV modeling, age distribution of pop validated against separate sources	None	None	None
**Recommendations**	Agent based models extended to include the effect of contact tracing, immigration; more data required to produce accurate estimates of transmission within households	Strategy of targeting TB control at HIV+ could be cost effect intervention; future modeling should incorporate improvement to social network modeling – either through graphing/ network or introduction of spatial relationships between households	Contact tracing is self- limiting in its cost effectiveness; individual based model with a network structure is next step	Interventions which treat as few as 5% of recent infections	Intervention which treats up to 40% of early LTBI
**Self-reported limitations**	Immigration, social risk factors and genetic risk factors not taken into account.	No cost effectiveness information for comparison with other interventions.	No age, contact structure or vaccination considered.	Does not account for HIV Preventative treatment may not be cost effective for certain targets.	Does not account for HIV High treatment rates for latent infection hard to achieve in practice as tracing majority of infections is difficult.

* Mean treatment duration assumed to be 0.5 years.

† Study cites previous study in which sensitivity analysis was conducted but did not repeat for this study [38].

### Setting of studies

Four of the studies presented models developed for low prevalence, developed world settings [19]–[22]. In these settings the background prevalence of latent TB infection is relatively low and health care systems frequently include contact tracing as a routine intervention for TB [19], [20]. Mellor et al. present a model for a high prevalence, developing world setting (Zimbabwe) where high HIV prevalence also contributes to the TB epidemic [23].

### Interventions investigated

Tian et al., Ziv et al. and Aparicio et al. [22] investigate the scope of contact tracing required to eliminate TB epidemics [20], [21]. Mellor et al. compare likely scenarios for implementation of contact tracing and high risk group investigations in a specific setting to compare their potential effects on incidence, prevalence, mortality and case detection rates [23]. Guzzetta et al. built a model designed to incorporate contact tracing but do not implement the intervention [19].

### Aims of the studies

All five studies primarily investigate model characteristics and appropriateness rather than focussing on projections or investigating the epidemiology of TB. For example, Guzzetta et al. and Mellor et al. investigate the improved fit to data and any increased insight provided by models with socio-demographic structure [19], [23]. However, all studies, except Guzzetta et al., did attempt to evaluate the effectiveness of contact tracing programs in a real world context [20], [21], [23].

### Models used

Models of infectious disease transmission are often categorised by features such as inclusion or exclusion of random effects (stochastic vs. deterministic) and the level of aggregation (compartmental vs. individual based models).

In deterministic models there is a fixed relationship between input parameters and model outputs so that the same set of initial conditions will always lead to the same outcomes [11]. Stochastic models allow some degree of chance at each time step leading to slightly different outcomes from each simulation. In compartmental models individuals in each class are only considered at an aggregate level where the importance of chance events, including in relation to interventions such as contact tracing, is usually ignored. Individual based models [24] instead simulate each individual in a population and allow for a more realistic implementation of targeted interventions such as contact tracing. These models are always stochastic in contrast to compartmental models which are typically (but not exclusively) deterministic and can be used to gain insight at a population level or for smaller groups of individuals [11]. Individual based models can provide an added level of realism that may provide more accurate insights into disease transmission and control measures. However, they often cannot be evaluated analytically and require considerably more computational power to analyse numerically than compartmental models. The increase complexity also typically leads to larger data requirements and other challenges in parameter estimation.

Tian et al.,Ziv et al. and Aparicio et al. [22] present deterministic compartmental models of TB transmission [20], [21] with uniform mixing in homogeneous populations. Mellor et al. present an individual based stochastic model with household-level clustering. The model incorporates increased individual contact within clusters but assumes that interactions between households are random, so that there is no wider community structure. Guzzetta et al. compare three different models of increasing complexity, starting with a homogenous compartmental deterministic model, followed by two individual based stochastic models which incorporate varying degrees of stratification, clustering and geographic complexity [19]. In their most complex implementation they include workplace and school clustering in addition to household clustering [19], [23]. Guzzetta et al. also assume random interactions outside of the specified clusters but in addition restrict these interactions spatially to emphasise contacts within nearby locations [19].

### Disease classes

The disease classes common to all five reviewed studies were susceptible, latently infected, and infectious. All of the studies except Aparicio et al. incorporated reactivation from latent infection into their models.Mellor et al. and Guzzetta et al. included relapse rates for recovered individuals [19], [23]. Aparicio et al. [22], Guzzetta et al. and Mellor et al. incorporate re-infection in their models and assume that latent infection or successful treatment/recovery provided some protection. None of the studies incorporated the Bacillus Calmette–Guérin (BCG) vaccination.

Tian et al. and Mellor et al. include a recovered class from which individuals may transition back to susceptible [25], [27], while only Ziv et al. assumed recovery from the disease provided full immunity against future disease [21]. Aparicio et al., Tian et al. and Mellor et al. include a treated class [20], [22], [23]. Mellor et al. and Ziv et al. include a breakdown of the latent class into early/late or fast/slow disease progression [21], [23]. Tian et al. subdivide classes by case detection status and along with Mellor et al. have classes for non-infectious active disease [20], [23].

For compartmental models such as that used in Tian et al., additional classes or compartments provide a more detailed method of incorporating contact investigation into the model. This has the potential to be more realistic than simply aggregating interventions into a detection or treatment rate but also causes a rise in model complexity. Individual based models are more readily adaptable to realistic implementations of contact tracing.

### Stratification

Risk factors such as age, gender, smoking or the presence of interacting infections such as HIV may also be taken into consideration in disease transmission models. Stratifying the simulated population by risk factor enables interactions between risk factors and transitions between disease classes to be incorporated. Key examples of where this may be important include rates of re-activation and the dependence of contact patterns on age, gender or location. Age is often a key determinant in disease transmission modelling as it strongly informs patterns of infectious contact. For many diseases age also influences susceptibility to infection or disease. In TB models the absence of age may result in an underestimation of transmission rates resulting in overestimation of reactivation rates [19], [26].

[TIGHER]Age stratification was implemented in Guzzetta et al. and Mellor et al. [19], [23] while Aparicio et al., Tian et al. and Ziv et al. implemented homogenous population structures [20]–[22]. Mellor et al. made use of age, not only directly in the TB model, but also as a means of determining the impact of sexual activity on HIV as a risk factor for TB [23]. Tian et al. suggest that the model they present would be improved by the use of age stratification and recommend future modelling efforts should include this feature [20].

While none of the models include the BCG vaccine, implementation of BCG in models is challenging due to variable estimates of its efficacy as well as its apparently differential impact on disseminated (and potentially fatal) disease as opposed to pulmonary (and hence transmissible) disease [27].

### Implementation of contact tracing

The practical conduct of contact tracing involves several steps, including identification of relevant contacts, decisions on the extent of tracing required and active recruiting of contacts for evaluation. Each step could be represented explicitly in models through the inclusion of additional classes and transition rates. Modelled steps would include probabilities of tracing any given contact, of contacts complying with testing, of tests detecting additional cases and the success probabilities of treatment – although these latter detection and treatment steps would also be required for regular passive detection with DOTS. Depending on the degree of complexity of the model being implemented some or all of these steps might be aggregated. For example an increased rate of detection could be implemented through an increased overall ‘treatment’ rate which incorporates both detection rates and subsequent treatment rates.

In the studies reviewed, contact tracing was implemented with varying degrees of realism. Aparicio et al. and Ziv et al. implement contact tracing at an aggregate population level in the form of increased preventative treatment rates for latently infected cases [21], [22]. Tian et al. include additional compartments for the population previously investigated through contact tracing or passive case detection [20]. Mellor et al. incorporate tracing of household level contacts using tests with specific detection parameters governing their accuracy (the tuberculin skin test (TST) and sputum microscopy) [23]. Guzzetta et al. while justifying their detailed community structure in terms of contact tracing, do not actually implement the intervention in their study [19].

### Comparisons with data

In an ideal situation modellers would have at least two independent data sets, where the first data set could be used for model fitting and parameter estimation and the second as a validation set to compare with model results [28]. However as data is typically limited modellers must often make a choice between the use of the available data for parameterisation or validation, or to use data from one data set for both purposes. Approaches taken in the reviewed papers are discussed below.

### Parameterization

The parameters of the model govern dynamic changes in disease classes through events such as disease transmission or recovery, as well as demographic processes such as births and deaths. All five studies made extensive use of literature to inform parameter values with additional estimation conducted through fitting models in all studies except Ziv et al. who took parameter values from previous work [21]. Epidemiological data used in parameter estimates was taken from a variety of sources including the WHO, CDC and other published studies. Mellor et al. made use of UNAIDS data and UK based sexual surveys in estimates of HIV prevalence [23]. Household surveys and census data were used for models incorporating heterogeneity in the population (age and/or household clustering) [19], [23].

Calibration of model parameters to data was attempted in four of the five papers, with Ziv et al. [21] choosing instead to use parameters taken from previous work. The method of calibration was different in each study, with maximum likelihood estimation used in Mellor et al. [23], polynomial least squares used in Aparicio et al. [22], a search optimization method in Tian et al. [20] and Latin-Hypercube samples combined with a least-squares threshold used in Guzzetta et al. [19] The source data for individual parameters in Tian et al. and Ziv et al. were not presented in their studies; Tian et al. list a number of sources for all parameter estimates while Ziv et al. state that estimates are taken directly from earlier work [20], [21].

The studies reviewed showed considerable variance in several key parameters (Table 1). For example, the number of new infections per infectious person per year varies between 7 and 18.8 infections while the mean duration of infectious period also varies from 0.3 to 2 years (Table 1). The mean survival time for TB cases also ranges from 3.3 years to 27 years. While setting dependent differences do occur in such parameters the variation in these key parameters is large and hinders model comparison. The models also incorporate treatment in different ways with Mellor et al. [23] and Tian et al. [20] using explicit detection and treatment rates while the other models use only aggregate treatment rates. When combined with the significant differences in parameter values described above, this variation makes parameter study outcomes difficult to summarise collectively. Choices in parameterization, both in terms of values and the structural form of the parameters, affect both the way interventions are implemented and their impacts with flow on effects to policy implications.

### Validation of model predictions

Baseline predictions (pre-intervention) were compared to observed epidemics to validate models in Aparicio et al., Guzzetta et al. and Mellor et al. [19], [22], [23] while Tian et al. present no baseline predictions [20] and Ziv et al. present models parameterized from previous work [21]. None of the reviewed studies attempt to validate the predicted efficacy of the contact tracing intervention with data. While there is little or no RCT data available to use in this instance, some observational data should be available in settings in which contact tracing has been in place as an intervention for some time (such as the USA) [29].

### Sensitivity Analysis

Aparicio et al. [22], Tian et al. and Mellor et al. discuss the sensitivity analysis conducted for the models they present [20], [23]. Aparicio et al. [22] vary the contact number (the number of secondary infections caused by an average case in a fully susceptible environment) through a realistic range. Tian et al. separately vary the number of contacts traced and mean time taken per investigation to determine how sensitive the model is to these parameters [20]. Mellor et al. find that their model is sensitive to reducing the long-term HIV prevalence, with reduced TB incidence the main impact (increases in this parameter had little effect) [23]. Ziv et al. do not perform sensitivity analysis for the model presented. Instead, they use estimates from previous work in which LHS was used in estimating parameters, but do not discuss sensitivity to this parameterization [21]. Similarly Guzzetta et al. use LHS in parameter estimation but do not discuss sensitivity [19].

### Key Findings of the Studies

Tian et al. demonstrate that for a specific low burden setting there are limited gains to be made from increased levels of detection beyond a certain point. They suggest that there is an optimal level that will result in eventual epidemic elimination beyond which there are diminishing returns from increased number of investigations [20]. Aparicio et al. and Ziv et al. find that effective treatment of latent TB cases (discovered through contact tracing) should result in TB epidemic elimination in a low burden setting [21], [22]. Mellor et al. find that targeting known high risk households with HIV positive individuals may be more effective than contact tracing [23]. Guzzetta et al. compare modelling methodologies for TB and find that socio-demographic individual based models provide good fits to available data and are of a form which allows evaluation of control strategies such as contact tracing [19].

### Recommendations of the Studies

The three recent studies recommend developing individual based models for the modelling of sophisticated social networks required for a detailed implementation of contact tracing [19], [20], [23]. Mellor et al. also suggests that targeted active case finding interventions in households with HIV-infected individuals may be more effective than contact tracing of TB-infected patients [23]. Tian et al. suggested that contact tracing is in a sense self-limiting and an optimal strategy could involve targeted investigations of intimate or close contacts, although their model does not provide information on the nature of contacts [25]. Aparicio et.al. and Ziv et al. suggest that using contact investigation programs to find recently infected persons may substantially contribute to the effort to control tuberculosis [21], [22], and Aparicio et al. go further to state that such a strategy may be more cost efficient than current strategies Guzzetta et al. [19] note the need for household transmission data to accurately estimate the impact of contact investigation interventions.

## Discussion

The papers reviewed in this study demonstrate alternative approaches to modelling contact tracing with differing potential to inform TB control policy. As Aparicio et al. and Ziv et al. [21], [22] illustrate, even a simple implementation of contact tracing through additional transition and rate parameters in a population-aggregated compartmental model has some utility in terms of the broad effects achievable through such a strategy. This approach requires assumptions to be made about the effectiveness of the intervention on a broad scale and avoids specifically modelling the interactions between individuals that give rise to infection risk. Models of this kind introduce contact tracing or other active case finding interventions with either increased detection rates or through further aggregation into an increased treatment rate. While the simplicity of this approach is advantageous for communication general predictions about contact-based strategies, such models are difficult to directly compare with data from TB control programs and cannot be used to address questions such as the optimal extent of contact tracing.

As contact tracing is necessarily an activity based around individuals, more detailed approaches require inclusion of individual characteristic in models. Elements which may need to be considered include incorporation of close as well as casual contacts, location-based clustering (such as within households, schools or workplaces), age-related associations, communal contact structures, and historical contact information. As more of these elements are incorporated into the study the choice of model structure must change to encompass them. For example by using a stochastic model with both individual and aggregate components Mellor et al. are able to include household clustering in their model [23] while Guzzetta et al. expand on this to incorporate clustering within households, schools and workplaces as well as spatial restrictions on the likelihood of contact based on commuting distance [19].

Each of the three recent papers [19], [20], [23] suggests moving to an individual-based modelling framework in which contact tracing can be simulated more directly. These allow the tracking and recording of interactions between individuals within an extended contact structure providing the potential to assess fine-grained variation in interventions and the effect of this detailed structure on outcomes. By directly tracking individuals in a population an increased level of realism regarding contact tracing intervention and disease transmission can be provided to policy makers who need to make decisions about implementing interventions. The individual based approach also has advantages in relation to modelling other components of TB disease including the complex natural history and characteristics relating to treatment completion.

However realism is not a virtue in and of itself and the additional complexity is only beneficial if it leads to an improvement in the accuracy and validity of model predictions. It is currently an open question as to what specific benefits individual based models of contact tracing will provide over simpler models as the reviewed studies have not yet implemented this approach in evaluation of interventions. However, there is clearly potential to improve the use of observational data in model fitting and validation through this approach.

While Guzzetta et al. [19] and Mellor et al. [23] make use of data to construct the social structure of their models, none of the reviewed studies use data to inform the effectiveness of contact tracing as an intervention. While the papers do not provide explanations for the omission of effectiveness data, it may relate to limited public availability of data from TB control units in settings where tracing is conducted. Modelling can be very valuable in the absence of high-quality data but to inform policy it is preferable for as much relevant data as possible to be used in order to produce accurate and robust predictions. Access to data on contact structures (such as household surveys) and on the effectiveness of contact tracing from trials and observational studies are likely to be particularly valuable. Setting specific data, where available, can often be essential in producing well calibrated models but also has the potential to mislead if applied out of context. For example, much of the reliable data available in the literature relates to declining European epidemics that were well-contained, while many models are being developed for high prevalence settings such as sub-Saharan Africa. Without setting-specific data, realistic predictions based on either direct or aggregate simulation of contact tracing interventions may not be achievable or of value to policy makers.

In general the availability of data should be a limiting factor on the level of complexity of a model. The models of Guzzetta et al. [19] incorporate a significant level of detail, however they still do so at the loss of a certain measure of realism, for example with the absence of immigration and vaccination programs. In the absence of specific data on household or workplace transmission rates this extra detail can introduce additional uncertainty, with additional assumptions required to generate simulations. Complex models can also be a barrier to translating findings into practice since they can be hard to explain and have computational demands that can limit analysis of robustness. Despite these challenges, complex models can also play a role in epidemiologic understanding by exposing influential processes which can be the subject of future field studies. In regard to contact tracing, it seems likely that development of individual based models will be required to improve understanding of the key factors underpinning its effectiveness.

Existing models used to address other questions in TB control including network based approaches [30] and alternative approaches to household clustering [31] have the potential to be adapted to assessment of contact tracing as an intervention. This approach has been used for other infections, such as Chlamydia, where transmission depends upon extended or repeated contact [32]. In low prevalence settings repeated close contact (often in the household or workplace) is the dominant cause of TB transmission. In this regard TB has similarities to STIs in which partners are usually known and can be traced [33],[34]. Techniques used to model these other diseases may be applicable to models of TB, but results or specific recommendations from these studies may be less transferable.

There are also a number of papers which attempt to develop broadly applicable implementations of contact tracing for use in deterministic models either directly or by approximation [25], [35]–[37]. These techniques may be applicable to TB models but are motivated by an intention to improve the implementation of contact tracing in commonly used deterministic models without requiring individual-based models. These approaches require less data than individual or network models while incorporating some features of more complex contact structures.

### Recommendations

Realistic implementations of contact tracing as an intervention in TB epidemics require the development of individual based models which can incorporate the required detail of contact structure. At present it is not clear that such approaches are superior to compartmental models as the value of this added realism will be best assessed through direct comparison between such models and simplified approaches.

The settings with the most potential to gain from implementing interventions such as contact tracing in addition to existing DOTS are those with high burdens from TB epidemics in which DOTS alone has not been sufficient to control the epidemic. There is an urgent need to develop models for settings both with and without high HIV prevalence which can be used to help make decisions about which interventions to put in place.

Access to and better use of more detailed data on the implementation and impact of contact tracing as an intervention, in addition to improved demographic data on contact and household structures are likely to be important elements of progress in this field. There is a great potential for collaborative work between clinicians, epidemiologists and modellers to use field studies and routine surveillance activities to gather the data required to inform future models.

## Conclusions

Models of contact based interventions have substantial potential to inform policy on TB control. Combined with cost effectiveness information models could be used to help make decisions about appropriate choice of interventions by comparing the relative costs and benefits of different strategies for contact tracing with other active case finding interventions (such as high risk group screening and mass radiography).

Although there is a large body of literature on TB transmission modelling there have been comparatively few models published which incorporate contact tracing as an intervention for TB. The existing studies we have reviewed offer insights into the potential benefits of contact tracing but are limited in detail and in context.

There is considerable scope for future analyses to make better use of data for model validation and to apply individual based models to facilitate more realistic patterns of infectious contact. A key focus of future modelling efforts should be to investigate the value of contact tracing as an intervention in settings with a high prevalence of TB (both with and without high HIV prevalence) or provide results which are generalizable to those settings. These settings are where the most gains can be made in global TB control.

## Supporting Information

Checklist S1 **PRISMA Checklist used in this study.**
(DOCX)
